# A novel CRISPR-Cas9 nickase-mediated rolling circle amplification (CRIRCA) technique for gene identification and quantitative analysis of extrachromosomal DNA

**DOI:** 10.1016/j.jare.2025.04.031

**Published:** 2025-04-22

**Authors:** Yuchen Song, Chaoyang Guan, Yue Zhang, Yiming Xu, Pengfei Li, Liqiang Luo, Chang Feng, Guifang Chen

**Affiliations:** a Center for Molecular Recognition and Biosensing, Shanghai Engineering Research Center of Organ Repair, Joint International Research Laboratory of Biomaterials and Biotechnology in Organ Repair (Ministry of Education), School of Life Sciences, Shanghai University, Shanghai 200444, PR China; b Department of Chemistry, College of Sciences, Shanghai University, Shanghai 200444, PR China; c Department of Applied Biology, East China University of Science and Technology, Shanghai 200237, PR China; d Shanghai Key Laboratory of Bio-Energy Crops, School of Life Sciences, Shanghai University, Shanghai 200444, PR China

**Keywords:** ecDNA detection, CRIRCA, CRISPR-Cas9, Tumor markers, Breast cancer

## Abstract

**Introduction:**

Extrachromosomal DNA (ecDNA) plays an important role in the initiation and progression of cancerous tumors. Although Circle-seq and other genetic technologies can be utilized for ecDNA analysis, they fail to provide multi-dimensional information from ecDNA, which is time-consuming and laborious.

**Objectives:**

Herein, by combining the netlike rolling circle amplification (NRCA) with CRISPR, we developed a novel CRISPR-Cas9 nickase-mediated RCA (CRIRCA) technology that can meet the clinical analysis needs of ecDNA.

**Methods:**

Atomic force microscope (AFM) was applied to confirm the circular structure of the ecDNA. Agarose gel electrophoresis was performed to analyze the CRIRCA products. Fluorescent detection was applied to characterize the fluorescence signal of amplified products. qPCR and FISH techniques were applied to verify the CRIRCA results of gene identification of ecDNA.

**Results:**

Our data revealed that CRIRCA achieved more efficient signal amplification compared to traditional RCA methods, allowing it to sensitively analyze small amounts of ecDNA in single tumor cells. Utilizing computer-aided design, we successfully constructed the primer library and sgRNA library of oncogene in ecDNA, and adopted CRIRCA technology to identify the oncogenes of ecDNA in breast cancer cells.

**Conclusion:**

Therefore, CRIRCA can simultaneously obtain the information from structure, sequence and quantitation of ecDNA. This work will fill the gap in the current research on the early monitoring of cancer targeting ecDNA, and provide support for the accurate diagnosis and treatment of cancer.

## Highlights

• A novel method ’CRIRCA’ was developed for the amplification and quantitative analysis of ecDNA by fusing CRISPR with NRCA.
• Sequence information, structural information, and quantitative information of ecDNA can be simultaneously obtained.
• CRIRCA can fill the gap in early cancer monitoring research targeting ecDNA, facilitating precise diagnosis and therapy.

## Graphical abstract

A novel nucleic acid amplification platform has been developed for the efficient analysis of extrachromosomal DNA (ecDNA) by fusing CRISPR with netlike rolling amplification (NRCA). This technology can simultaneously obtain sequence information, structural information, and quantitative information of ecDNA.

**Figure:**
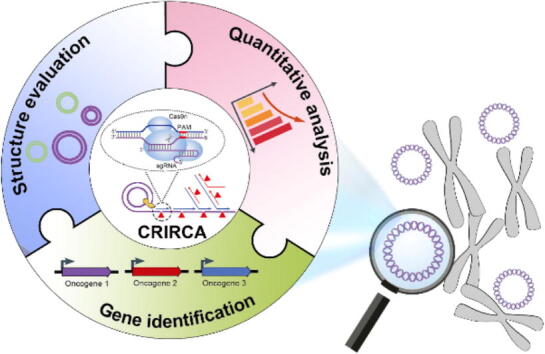

## Introduction

Extrachromosomal DNA (ecDNA) refers to a type of circular DNA that is found in eukaryotes outside the chromosomes [1,2]. Due to chromothripsis and apoptosis, specific nucleases will cleave genomic DNA in the interstellar region of nucleosomes, resulting in the shedding of linear DNA fragments from the original chromosomes and being cyclized by DNA ligase in eukaryotic cells [[3], [4], [5]]. The circular ecDNA carries a large number of oncogenes, and some long ecDNA segments even carry the complete sequence of an oncogene [[6], [7], [8]]. Additionally, the presence of circular ecDNA drives the expression of oncogene, resulting in an abnormal increase in oncogene expression and also making cancer cells prone to heterogeneity [[9], [10], [11]].

At present, the analysis of ecDNA primarily relies on circular DNA sequencing (CIRCLE-seq) technology and traditional genetic analysis [12,13]. The former is based on the rolling circle amplification (RCA) technology, which utilizes the differences in the structure and chemical properties of linear DNA and circular DNA. The information regarding ecDNA is obtained through high-throughput sequencing of the amplified products [14,15]. However, the sensitivity of this technology is currently insufficient for quantitative analysis of ecDNA. Furthermore, due to the relatively low abundance of ecDNA in cells, a large number of cells (approximately 1 × 10^7^) are required, making it challenging to apply for single-cell analysis and heterogeneity analysis [16,17]. In addition to the challenges mentioned earlier, it is worth noting that this technology also requires expensive sequencing equipment and rigorous signal processing, which significantly contribute to the overall high operational costs [18,19]. The latter utilizes traditional genetic analysis, specifically karyotype analysis, which involves observing chromosomes through microscopes to detect the presence of ecDNA [20,21]. However, this technology not only requires the utilization of costly high-resolution imaging equipment but also fails to obtain the sequence information of ecDNA [22].

On the other hand, the analysis of ecDNA for clinical diagnosis and treatment remains underexplored and lacks sufficient research. Above techniques and other current related techniques face challenges in their application due to the necessity of taking into account both the structure, sequence, and quantitative information of ecDNA in clinical applications [23]. In terms of structure, due to the significantly larger quantity of chromosomal DNA compared to ecDNA, it is challenging to distinguish between ecDNA and chromosomal DNA without information regarding the circular structure [24,25]. In terms of sequence, it is crucial to determine whether the ecDNA present in clinical samples carries oncogenes, as well as the specific type of oncogenes it carries. In terms of quantification, the amount of ecDNA plays a crucial role in indicating the level of gene expression, which can provide valuable insights into the degree of malignancy and developmental status of cancer cells [26,27]. Although the above-mentioned techniques of ecDNA analysis provide intuitive structural information and in-depth sequence information respectively, they may be challenging to meet the cost control and operability requirements for clinical diagnosis and treatment.

In order to fill the blank in the clinical research of ecDNA, we have developed the CRISPR Cas9 nickase mediated RCA (CRIRCA) technology by combining netlike rolling circle amplification (NRCA) with the highly promising gene-editing tool, CRISPR. NRCA is a novel RCA technology that we previously developed by building upon the principles of traditional RCA and hyperbranched RCA (HRCA). This advancement was achieved through the strategic incorporation of nicking enzymes and the rational design of sequences that specifically cut the HRCA products. NRCA enables three-dimensional signal amplification, significantly enhancing amplification efficiency. However, NRCA is dependent on site-specific nicking enzymes, which impose limitations when analyzing ecDNA sequences that are long and structurally complex [[28], [29], [30], [31], [32], [33], [34]]. In this innovative CRIRCA method, by utilizing ecDNA as a circular DNA template, we can employ isothermal amplification to generate long DNA fragments with repetitive complementary sequences, which indirectly provides valuable information about the circular structure of the ecDNA that can be distinguished from linear chromosomal DNA based on the presence of disperse electrophoretic bands [35]. Moreover, the advanced specificity and versatility of CRISPR technology allow for the design of specific guide RNA (sgRNA) tailored to the target sequence, which helps us eliminate the limitations associated with the specific site requirement of nicking enzymes [36]. By designing primer libraries for multiple oncogenes, the CRIRCA assay can initiate amplification reaction on the basis of complementary binding between ecDNA and specific primers, which affords comparable performance in gene identification compared with qPCR. Furthermore, CRIRCA simultaneously harness both NRCA and nick-cleavage activities of the CRISPR-Cas9n system, which enable cascade signal amplification of ecDNA, allowing for accurate and quantitative analysis of ecDNA. In comparison with CRISPR-CATCH, which focuses on specific ecDNA region enrichment through CRISPR-Cas9-based cleavage and pulse-field gel electrophoresis, CRIRCA offers a more straightforward, high-sensitive approach for detecting a wide range of ecDNA sequences without the need for pre-existing sequence knowledge [16]. Hence, our proposed CRIRCA fusion technology holds the potential to simultaneously acquire comprehensive structural, sequence, and quantitative information from ecDNA, which is expected to greatly facilitate the integration of ecDNA detection into clinical practice.

## Materials and methods

### Reagents and apparatuses

All oligonucleotides (Table S1) were provided by Sangon Biotech. Co., Ltd. (Shanghai, China). Klenow fragment (exo-) and Nt.BsmAI were all purchased from New England Biolabs (USA). Pyrophosphatase, Inorganic (yeast) (YIPP) was supplied by New England Biolabs (USA). Plasmid-Safe™ ATP-Dependent DNase (E3101k) was purchased from Lucigen (USA). FastDigest MssI (GTTT**^**AAAC) was purchased from Thermo Scientific (USA). HiSpeed Plasmid Midi Kit was purchased from Qiagen (Germany). Agarose was purchased from Baygene Biotech Co., Ltd. (Beijing, China). 5 × loading buffer, gel extraction kit and BCA protein assay kit were purchased from Generay Biotech (Shanghai, China) Co., Ltd. Deoxynucleotide triphosphates solution mixture (dNTP) mixture was supplied by Sangon Biotech. Co., Ltd. (Shanghai, China). 100 bp and 250 bp-Ⅱ DNA ladder were purchased from Generay Biotech (Shanghai, China) Co., Ltd. SYBR Green I (10000×) was purchased from Solarbio (Beijing, China). Hieff qPCR SYBR Green Master Mix was purchased from YEASEN (Shanghai, China). HyperScribe T7 high yield RNA synthesis kit was purchased from APExBIO Technology (USA). 10 × TAE buffer (Tris-acetate-EDTA) and 10 × PBS buffer were purchased from Ourchem (Shanghai, China). DMEM basic (1×), RPMI Medium 1640 basic (1×), Leibovitz's L-15 Medium, Mammary Epithelial Cell Medium and Fetal Bovine Serum (FBS) were purchased from Gibco (USA). 0.25 % Trysin-EDTA (1×) was purchased from BI (Israel). MCF-10A, BT474, T47D, MDA-MB-231 and MCF-7 were purchased from HATAKA (Japan). All chemical reagents used in this experiment were of analytical grade. All solutions were prepared using Milli-Q water at 18.2 MΩ•cm^−1^, obtained from a Milli-Q purification system (Millipore, USA).

### Extraction and enrichment of ecDNA

To maximize extraction efficiency, 4 × 10^7^ cells were initially used, as recommended by the HiSpeed Plasmid Midi Kit and supported by relevant literature [37]. The cells were collected by centrifugation at 1000 rpm for 15 min at a temperature of 4 °C. Cells were then resuspended in Buffer P1 containing RNase A. For lysis, Buffer P2 was added, and the tube was inverted vigorously 4 to 6 times, followed by a 5-min room temperature incubation. Subsequently, Buffer P3 was introduced to the lysate, mixed immediately by inverting the tube 4 to 6 times. A HiSpeed Midi Tip was prepared by applying 4 mL of Buffer QBT, allowing gravity to facilitate flow. The cell lysate was then filtered through a QIAfilter Midi Cartridge into the equilibrated HiSpeed Tip. Following this, the HiSpeed Tip was washed with Buffer QC. DNA was then eluted using Buffer QF and precipitated by the addition of isopropanol. The mixture containing isopropanol was transferred to a syringe and passed through the QIAprecipitator by using the plunger. The DNA, now in the QIAprecipitator, was washed with 70 % ethanol, followed by elution into a collection tube using 1 mL of Buffer TE through a 5 mL syringe. This process yielded crude ecDNA.

In the exonuclease treatment, 40 μL crude ecDNA, 4 μL of FastDigest Buffer (10×) and 1 μL of FastDigest MssI were added to linearize mitochondrial DNA (mtDNA). The procedure involved an initial incubation at 37 °C for a duration of 10 min, followed by a subsequent 10-min incubation at 65 °C to cease the reaction. After this, the mixture received 10 units of Plasmid-Safe™ ATP-Dependent DNase, along with 4 μL of 10 × DNase buffer and 2 μL of 25 mM ATP, integrated into the digestion system. Then, the above reaction was incubated for 120 min at 37 °C. To stop the digestion process, a final incubation step was carried out at 70 °C for 30 min.

### Atomic force microscope (AFM) characterization of ecDNA

AFM was confirmed to the circular structure of ecDNA, as its circular form is crucial for distinguishing it from linear chromosomal DNA. A mixture was prepared by combining 10 μL of the purified extrachromosomal circular DNA (ecDNA) with 10 μL of a 20 mM MgCl_2_ solution, which was then applied to a mica slice supplied. This setup was left to incubate at ambient temperature for 10 min. Subsequently, the surface of the mica slice was gently washed with water for 30 s and then dried thoroughly using nitrogen gas. For morphological analysis of the ecDNA on the mica, an *ex situ* Agilent 5500 AFM was employed. The scanning was done in tapping mode at a rate of 0.5–––1 Hz, with the resonant frequency of AFM tips ranging between 160–––260 kHz. Images were captured at a resolution of 512 × 512 pixels.

### Cas9n (D10A) protein expression and purification

Cas9n(D10A) protein expression vectors were transformed into BL21(DE3). A 5 mL starter culture was grown overnight in Luria-Bertani medium (LB), which was used to inoculate 200 mL of LB for growth at 37 °C and 200 rpm until an OD_600_ of 0.6. At this time, 0.06 % 200 mg/mL IPTG was introduced to induce protein expression, and the culture was continued at 16 °C, 200 rpm for 16 h for protein expression.

Cells were then centrifuged at 8000 rpm for 3 min at 4 °C and cell pellet was crushed and resuspended in Purification Buffer (50 mM Tris-HCl, 300 mM NaCl, pH 7.5) by sonication (SCIENTZ Ultrasonic Homogenizer JY29-IIN) with the following conditions: power of 130 W for 2 s on and 2 s off with a total sonication time of 4 min. Lysate was divided into supernatant and precipitation by centrifugation at 12000 rpm, for 30 min at 4 °C.

Affinity chromatography using a Ni-NTA medium was utilized to purify the lysate's supernatant. The process of eluting proteins involved a series of buffers: Purification Buffer, followed by a graded series of Elution Buffers. These included Elution Buffer I with a composition of 50 mM Tris-HCl, 300 mM NaCl, and 20 mM imidazole at pH 7.5, and subsequent buffers (Elution Buffer II to Elution Buffer VII) increasing the concentration of imidazole from 50 mM to 500 mM, all maintaining the same pH and Tris-HCl and NaCl concentrations. The fractions with higher protein concentrations were then combined and subjected to dialysis against a Storage Buffer, composed of 50 mM Tris-HCl, 300 mM NaCl, 1 mM DTT, and 20 % glycerol at pH 7.5. The dialysis products were centrifuged at 5000 rpm for 15 min on microseps, repeating 3 times to concentration proteins. The final concentration of Cas9n(D10A) is 8.48 μM.

### Computational design of the sgRNA and primer libraries

The target gene sequences, including EGFR, EZH2, HER2, and Bt, were retrieved from the NCBI GenBank database. Primer libraries were computationally designed using Primer Premier 6.0 software, with critical parameters optimized for experimental requirements, including primer length (18–22 bp), melting temperature (Tm: 55-65°C), GC content (40–60 %), and primer compatibility. Target-specific regions were selected for primer design through both automated algorithms and manual refinement to ensure optimal binding characteristics. All designed primer pairs were subsequently subjected to specificity validation using the NCBI BLAST online tool, confirming their unique binding to the intended genomic loci.

For sgRNA library construction, we employed CRISPR design tools available through the Addgene platform, specifically utilizing CHOPCHOP and CRISPOR algorithms. Target gene sequences were input into these platforms to generate optimized sgRNA candidates based on predicted on-target efficiency and off-target effects. The selected sgRNAs were then synthesized through *in vitro* transcription, followed by quality control assessment, to establish a comprehensive sgRNA library for gene identification using CRIRCA.

### In vitro transcription of single guide RNA (sgRNA)

DNA templates were prepared by mixing 10 μM sense and antisense strands of the template, which were denatured at 95 ℃ for 10 min, and then gradient annealed to 25 ℃ to synthesize double stranded DNA template. Then, *in vitro* transcription reaction system containing 2 μL of DNA templates, 2 μL of ATP, GTP, CTP, and UTP (20 mM), 2.5 U/μL of T7 RNA polymerase, 2 μL of 10 × T7 RNA Buffer and 6 of μL DEPC water with a total reaction volume of 20 μL were incubated at 37 ℃ for 4 h to synthesize sgRNA.

### Plasmid DNA cleavage experiment

Plasmid DNA was used to analyze the feasibility of CRIRCA before the use of ecDNA. First, 100 nM Cas9n and 200 nM sgRNA were mixed and incubated at 37 ℃ for 30 min in NEBuffer 3.1. Then, 10 nM pUC57 plasmid was added to the sgRNA: Cas9n complex. The cleavage reaction mixture was incubated at 37 ℃ for 60 min and characterized by 1.5 % agarose gel electrophoresis.

### NRCA reaction

To evaluate the performance of other methods based on RCA in amplifying circular DNA templates. The plasmid amplification was carried out using the NRCA method in a 50 μL reaction. This reaction included 50 ng of pUC57-hGluc (stored in our laboratory), primers 1 and 2 each at 200 nM, 200 μM of dNTPs, 1 × NEBuffer2, 2.5 U of YIPP, 15 U of Klenow polymerase, and 5 U of Nt.BsmAI. The NRCA was performed at 37 °C, followed by a final incubation at 75 °C for 15 min to stop the reaction. For HRCA and RCA protocols, modifications were made: Nt.BsmAI was omitted from the HRCA, and both primer 2 and Nt.BsmAI were excluded from the RCA process.

### CRIRCA reaction

The reaction mixtures for CRIRCA were prepared with 50 ng of purified ecDNA, 200 nM primers (random primers for quantitative analysis, primer 1 and primer 2 for gene identification), 200 μM dNTPs, 1 × NEBuffer2 and ddH_2_O to make total volume reached 50 μL. The CRIRCA procedure was conducted using a ThermoStat plus (Eppendorf, Germany), initially heating to 95 °C for 10 min, followed by a 5-min annealing phase at 25 ℃. Subsequently, the reaction mixture was supplemented with 2.5 U of YIPP, 15 U of Klenow polymerase, Cas9-nickase at 100 nM, and 200 nM of sgRNA. This mixture was then incubated at 37 °C, with a final 15-min incubation at 75 °C to conclude the reaction. It is important to note that Cas9n and sgRNA were pre-incubated together at 37 °C for 30 min to facilitate the formation of sgRNA: Cas9n complexes. As for RNA, both Primer 2 and the sgRNA: Cas9n complexes were excluded. As for hyperbranched rolling circle amplification (HRCA), sgRNA: Cas9n complexes was removed, while other conditions remained unchanged.

### Gel electrophoresis analysis

Electrophoretic analysis of ecDNA, plasmids, and CRIRCA products was carried out using 1 % agarose gel. For this, a mixture of 4 μL DNA samples and 1 μL of 5 × loading buffer, which includes Gelred nucleic acid dye, was prepared. Additionally, 2 μL of the 100 bp DNA ladder was loaded for reference. The electrophoresis was performed at 120 V for 30 min in a 1 × TAE buffer system. After electrophoresis, the Bio-Rad GelDoc XR system was employed to capture and analyze the band patterns on the gel, facilitating the analysis of the results.

### Fluorescent detection

1 μL 1000 × SYBR Green I and 50 μL HRCA and CRIRCA products were mixed and incubated at room temperature for 10 min. F-7000 fluorescence spectrometer was used to characterize the fluorescence signal of amplified products. The excitation wavelength was 490 nm and the emission were measured in the range from 500 nm to 650 nm with a slit width of 5 nm and an excitation photovoltage of 750 V. The fluorescence intensity was recorded at the emission wavelength of 520 nm.

### qPCR

qPCR was used to quantitatively validate the gene identification results obtained from CRIRCA. Primers for qPCR were specifically designed to target the oncogene sequences present in ecDNA. The qPCR assay was conducted in a reaction volume of 25 μL, which included 2 μL of ecDNA as the template, 1 μL each of 5 μM primers 1 and 2, 12.5 μL of 2 × qPCR Mix, and 8.5 μL of ddH_2_O. The qPCR was performed using the CFX-96 Real-Time System (Bio-Rad, USA) with the following thermal cycling conditions: an initial denaturation at 95 ℃ for 5 min, followed by 40 cycles of (95 ℃ for 30 s, annealing temperature for 30 s, and 72 ℃ for 30 s). This reaction was terminated with a final extension step at 72 ℃ for 10 min and a holding period at 25 ℃. The annealing temperatures of EGFR, EZH, HER2 are 54 ℃, 54 ℃, 61 ℃, respectively.

### Fluorescence in situ hybridization (FISH)

The FISH experiment was to confirm whether the selected oncogenes located on ecDNA are present in the nuclei of cancer cells. Breast cancer cells in the logarithmic proliferation phase were digested and collected with trypsin. 5 mL of 0.075 M KCl was added to the cell precipitation, gently blown and mixed, and incubated at room temperature for 30 min. 1 mL of fixative (methanol: glacial acetic acid = 3: 1) was added to the cells, gently blown and mixed, and centrifugated at 1200 rpm for 5 min. After removing the supernatant and adding 5 mL of fixative, the cells were fixed for 20 min at room temperature and then centrifuged at 1200 rpm for 5 min. By removing the supernatant, the cells were resuspended with 1 mL of fixative and stored at −20 ℃. 2–3 drops of cell suspension were added on the pre-cooled clean slide. Then the slide was passed through the alcohol lamp flame several times and dried at room temperature. The slide was placed at 2 × SSC for 2 min, soaked in 70 %, 85 % and 100 % ethanol at room temperature for 2 min respectively, and then dried at room temperature. 10 μL FISH probe was added to the slide sample site and covered with 22 × 22 mm cover glass. Then, the FISH slides were incubated at 85 ℃ for 5 min and incubated at 37 ℃ for 16 h. After removing the glue on the glass, the glass slide was washed at 74 ℃ for 2 min, and then transferred to room temperature for 2 min. The slides were dehydrated with 75 %, 85 % and 100 % alcohol for 1 min, and then dried in air. 20 μL DAPI was added to stain the cell nucleuses for 15 min. Fluorescence images were obtained using a LSM 710 confocal laser scanning microscope.

## Results and discussion

### The extraction and characterization of ecDNA

**Fig. 1:**
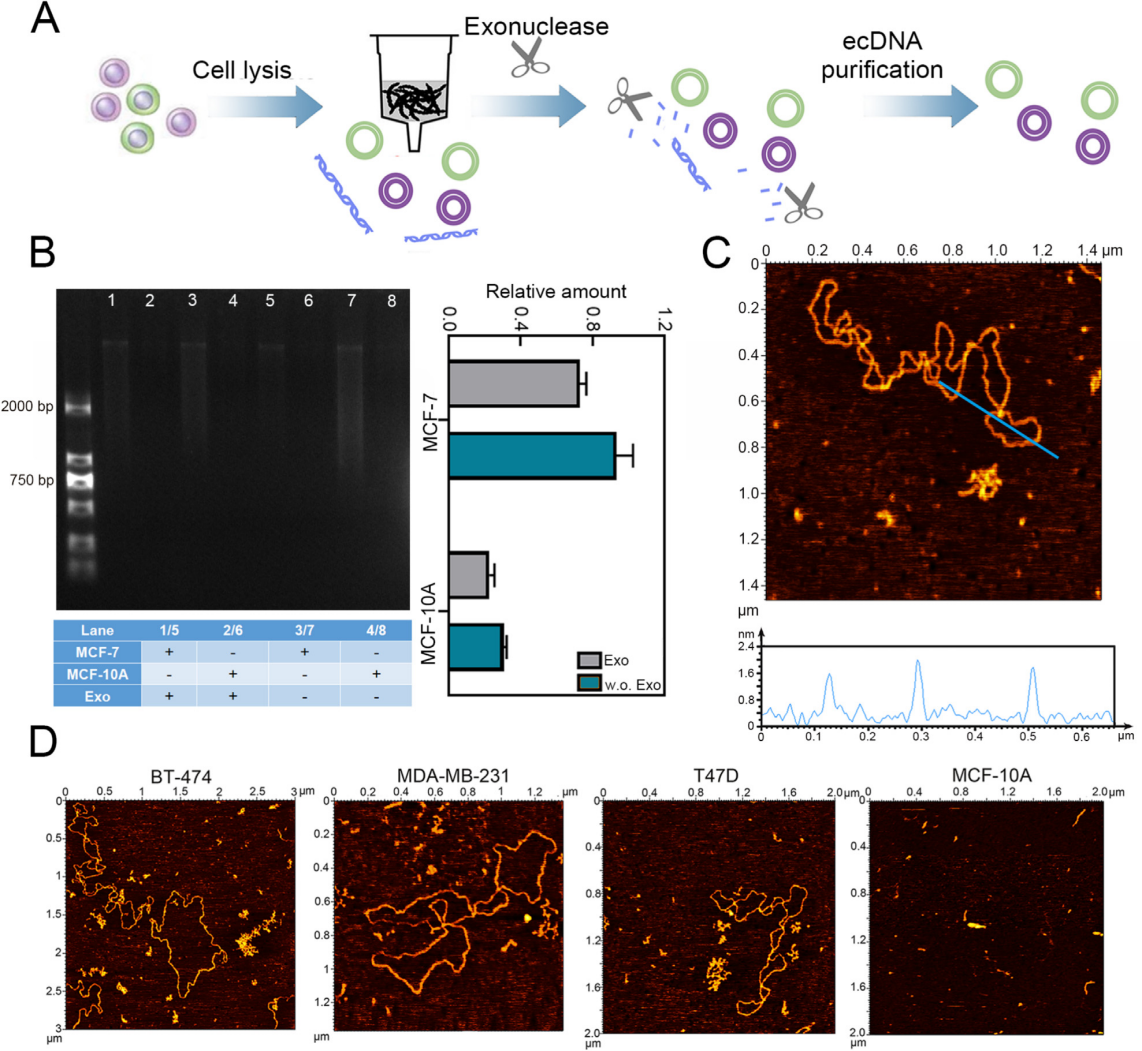
Verification of extraction of ecDNA. (A) Schematic diagram of isolation and purification of ecDNA. (B) Electrophoretic characterization of ecDNA extracted from MCF-10A and MCF-7 cell. Exo represents ecDNA with ExoI treatment, w.o. Exo represents ecDNA without ExoI treatment. (C) AFM image of purified ecDNA extracted from MCF-7 cell. The height of the positions marked with blue lines is shown under the corresponding AFM image. (D) AFM images of purified ecDNA extracted from BT-474, MDA-MB-231, T47D and MCF-10A cells. (For interpretation of the references to colour in this figure legend, the reader is referred to the web version of this article.)

Using the strategy of plasmid extraction, we extracted ecDNA from tumor cells by alkaline extraction. Meanwhile, nucleic acid tool enzymes are also used to digest residual linear DNA to obtain ecDNA with higher purity (Fig. 1A). The extracted ecDNAs were characterized by agarose gel electrophoresis before and after being treated with exonuclease. The electrophoresis result showed that the band of ecDNA (lane 2/6) of MCF-10A digested by exonuclease was almost invisible, whereas the purified ecDNA bands of MCF-7 were faintly visible (lane 1/5). At the same time, the gray value analysis results of electrophoresis bands showed that the purified ecDNA content extracted from MCF-7 cells was higher than that of MCF-10A cells (Fig. 1B). In addition, we further confirmed the circular structure of the purified ecDNA using AFM. It could be seen that the structure of ecDNA were circular, and the height were about 1.6 nm, which conformed the characteristics of double-stranded DNA (Fig. 1C). We also extracted and purified ecDNA from other breast cancer cell lines (BT-474, MDA-MB-231and T47D), and their circularity were the same as that of MCF-7. It is worth mentioning that the purified ecDNAs extracted from normal cells are much less than that in tumor cells. (Fig. 1D).

### Verification of CRIRCA for ecDNA detection

**Fig. 2:**
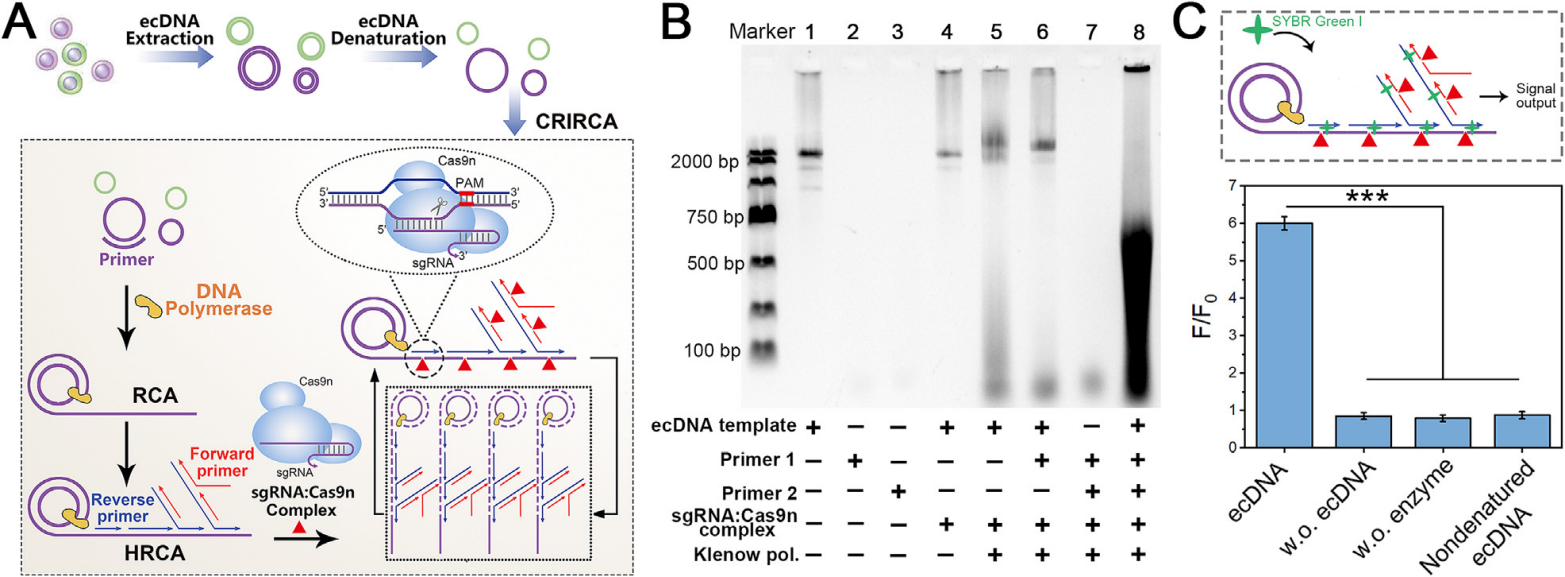
Feasibility of CRIRCA for ecDNA detection. (A) Schematic of the proposed CRIRCA for amplification of ecDNA. (B) Agarose gel electrophoresis analysis of the feasibility of CRIRCA. (C) Fluorescence verification of CRIRCA. ecDNA represents complete CRIRCA system including ecDNA template, primer1, primer2 and enzymes. w.o. ecDNA represents ecDNA-free control including primer1, primer2 and enzymes. w.o. enzyme represents enzyme-free control including ecDNA template, primer1 and primer2. nondenatured ecDNA represents native ecDNA system including nondenatured ecDNA, primer1, primer2 and enzymes. Standard deviations are represented by the error bars based on data from three replicates. (n = 3, ***p < 0.001).

CRIRCA technology integrates NRCA and CRISPR technology, the principle of which is shown in Fig. 2A. First, extracted ecDNAs are denatured to form a single strand. Then primer 1 binds to single-stranded circular ecDNA and initiates rolling circle amplification (RCA) [38]. Next, primer 2 could be complementary to the extension product of primer 1, and further undergo branch strand extension and strand displacement reaction by DNA polymerase, which is named hyperbranched rolling circle amplification (HRCA) [39]. Cas9n is an enzyme with endonuclease activity formed after the modification of Cas9 protein. Unlike ordinary endonuclease, its cleavage site needs to be guided by the corresponding sequence of sgRNA. Based on the specific sequence distribution of ecDNA, we designed corresponding sgRNAs, which recognize and cleave specific sites of the circular template to induce a CRIRCA reaction. In the presence of Cas9n, the HRCA products at designated sites undergo specific recognition and cleavage into shorter fragments. These fragments then act as novel primers, instigating additional HRCA reaction. For assessing the amplification efficiency, the reaction mixtures were supplemented with Sybr Green I (at a concentration of 1000×). This cycle of primer extension, strand displacement, and nicking facilitated by the Cas9n-assisted amplification process, enabling sensitive detection of low abundance ecDNA.

In order to verify the feasibility of CRIRCA for the detection of ecDNA, we first adopted RCA, HRCA and NRCA technology to amplify circular plasmid DNA, the structure of which is similar to that of ecDNA. The selected plasmid DNA is pUC57 artificially constructed to express humanized gaussia luciferase with a length of 3359 bp (Fig. S1A). We have used the same extraction method to extract approximately the same amount of bacterial plasmid and analyze the morphology and structure of the plasmid. As shown in Fig. S1B, the circular structure of plasmid DNA can be seen in the AFM image, proving that the method has successfully isolated and enriched plasmid DNA. Then we used RCA, HRCA, and NRCA methods to amplify plasmid DNA. The feasibility and kinetics analysis in Fig. S1C-D also indicated that the amplification efficiency of NRCA is higher than that of HRCA and RCA derivative techniques can amplify double stranded circular templates, which provides a feasible basis for CRIRCA technology to amplify ecDNA. Moreover, fluorescence spectral analysis was conducted for the NRCA reaction to compare various concentration of plasmids. Fig. S1E shows that when the target concentration changes from 70 fM to 10 pM, the fluorescence value shows a good logarithmic linear relationship with the target concentration. The linear equation is Y = 0.094X + 0.1 (R^2^ = 0.995), with a detection limit of 7.24 fM. In addition, we have employed AFM to successfully characterize the morphology of the HRCA products for plasmid as shown in Fig. S1F, which present corresponding hyperbranched appearances. The above results showed that RCA and its derivatives could meet the requirements of amplification of circular DNA with long sequence.

Next, we integrate the CRISPR system with the NRCA reaction by expressing Cas9 nuclease with nickase activity. The Cas9n with HNH activity, which refers to the nicking activity of the HNH domain, was obtained by introducing the D10A mutation (Asp^10^ → Ala^10^) in the catalytic residues of the RuvC domain. This mutation inactivates the RuvC domain, while retaining the ability of HNH domain to generate a single-strand break (nick) in the target DNA. The construction process of plasmid and protein purification were shown in Table S2-S5 and Fig. S2-S4, and the results showed that the Cas9n was successfully constructed. Moreover, the nickase activity of Cas9n was verified by agarose electrophoresis analysis of pUC57 plasmid cleavage by sgRNA:Cas9n complexes (Fig. S5). The result indicated that the sgRNA:Cas9n complexes displayed active HNH domain which specifically nicked one strand of plasmid DNA showing open circular structure. Then, to examine the feasibility of CRIRCA for ecDNA detection, the amplified products of ecDNA were analyzed and characterized using agarose gel electrophoresis (Fig. 2B). In the presence of sgRNAs:Cas9n complex (lane 4), the band of ecDNA (lane 1) became faint and a new band near the hole was observed, suggesting that the ecDNA was cleaved to generate nicked DNA. When the Klenow polymerase and primer 1 were present (lane 6), the disperse band appeared, indicating that primer 1-assisted priming, extension, and nicking occurred. When the primer 1 and primer 2 were both present, the blocked band and disperse band were brighter compared to lane 6, showing the obvious characteristics of the products amplified by the circular template, which can distinguish ecDNA from linear chromosomal DNA. Additionally, fluorescence analysis was carried out to validate the CRIRCA for ecDNA detection, which was though adding fluorescent dye (syber green I) as signal output (Fig. 2C). Compared with negative control groups significant fluorescent signal was observed when the target ecDNA was present, which also confirm the feasibility of the construction of CRIRCA system. The above results confirm the feasibility of our proposed method for the amplification of ecDNA. Additionally, we assessed the potential impact of linear DNA that might remain after the Plasmid-Safe DNase treatment on CRIRCA, and the results showed that the fluorescence signal was significantly lower (about 3-folds) when the enzyme was used (Fig. S6).

### Optimization of CRIRCA reaction conditions

**Fig. 3:**
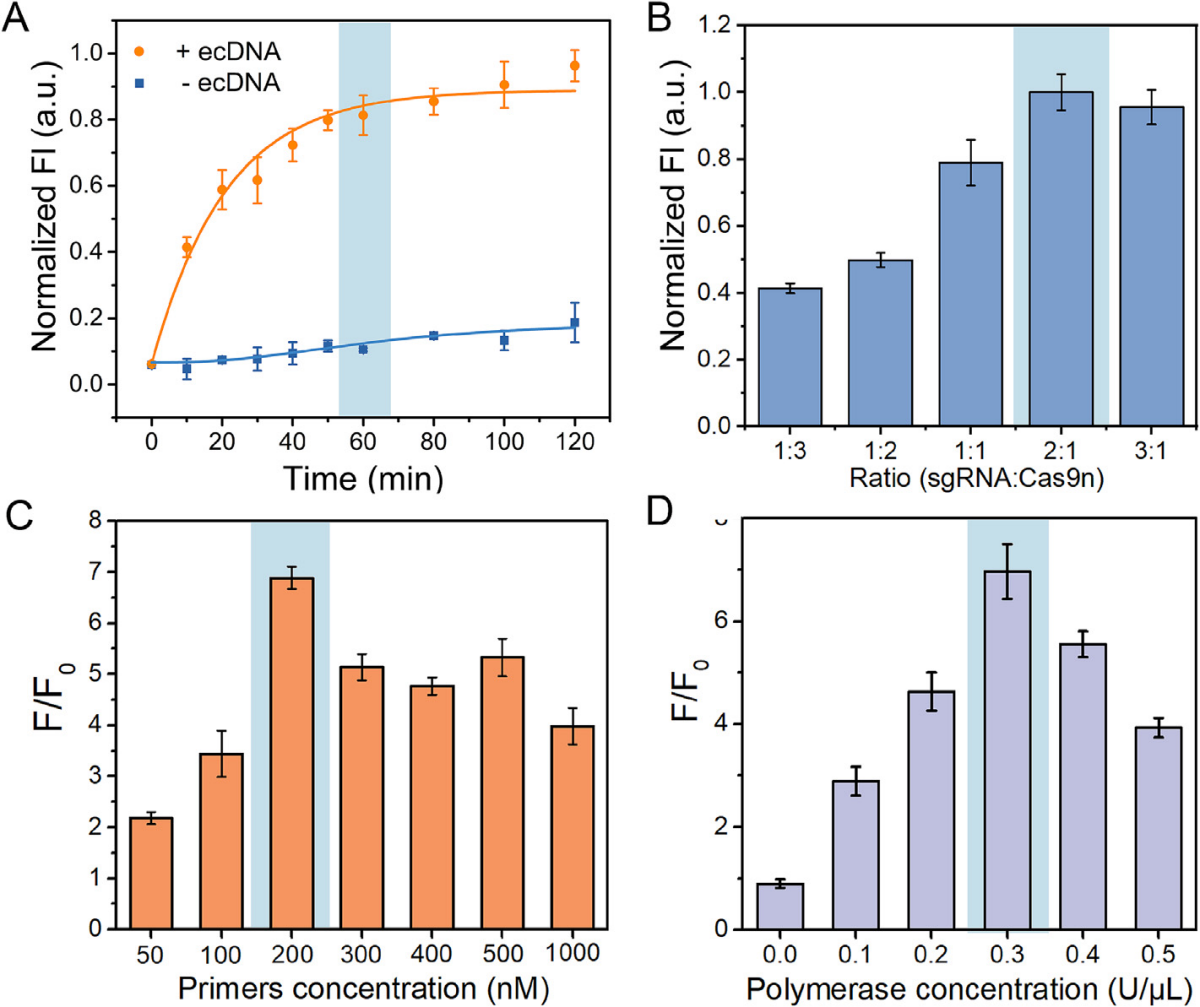
Evaluation of the optimal reaction conditions of CRIRCA. (A) The fluorescence curve of CRIRCA at different reaction time. (B) Fluorescence intensity of target amplification products at different concentration ratio of sgRNA and Cas9n. (C) Fluorescence signal-to-noise ratio of CRIRCA at different concentration of primer1 and primer2. (D) Fluorescence signal-to-noise ratio of CRIRCA at different concentration of Klenow polymerase. Error bars represent standard deviations from three replicates.

We further evaluated the possible factors that may affect the CRIRCA reaction for detecting ecDNA. The optimal reaction time was investigated through monitoring the reaction kinetic process within 2 h. In the absence of target ecDNA, the upward trend of fluorescence intensity is weak, which suggested that CRIRCA exhibits good specificity. In the presence of target ecDNA, the fluorescence signal gradually increased and showed significant difference compared with the negative control. As the reaction approached 60 min, a gradual plateauing of the fluorescence signal was observed. Consequently, 60 min was determined to be the most suitable reaction time for the CRIRCA process. (Fig. 3A). Moreover, Cas9n needs to be guided by sgRNA to exhibit cleaving ability. Therefore, we explored the effects of different concentration ratio of sgRNA and Cas9n on amplification efficiency. When the ratio of sgRNA and Cas9n was 1:3, 1:2 and 1:1, the fluorescence signal was relatively low, indicating that Cas9n cannot fully exert its function when sgRNA is insufficient. When the ratio of sgRNA and Cas9n was 2:1, fluorescence signal obviously enhanced, which suggested that only when the amount of sgRNA was relatively excessive, the cleaving effect of Cas9n was sufficient to fully display. Therefore, 200 nM sgRNA and 100 nM Cas9n were selected as the optimal reaction concentration (Fig. 3B). Considering primers concentration is one of the key factors for non-specific amplification, then we investigated the effect of primer concentration on CRIRCA. As shown in Fig. 3C, the fluorescence signal-to-noise ratio gradually increased as the primer concentration increased. When the concentration of the primer is 200 nM, the fluorescence signal-to-noise ratio reaches its highest value. Whereas the primer concentration is higher than 200 nM, the fluorescence signal-to-noise ratio gradually decreased. We speculated that high concentration of primers can lead to uncertain secondary structures between primers, leading to nonspecific amplification. Therefore, 200 nM was chosen as the optimal primer concentration for CRIRCA. The concentration of polymerase is also the key factor in nucleic acid amplification. The fluorescence signal-to-noise ratio of CRIRCA at different concentration of Klenow polymerase was measured as shown in Fig. 3D. The signal-to-noise ratio increased with the increase of polymerase concentration. When the concentration exceeded 0.3 U/μL, the amplification efficiency gradually decreased as the concentration increased. Therefore, 0.3 U/μL was selected as the optimal concentration of Klenow polymerase in CRIRCA system.

### Quantitative analysis of ecDNA

**Fig. 4:**
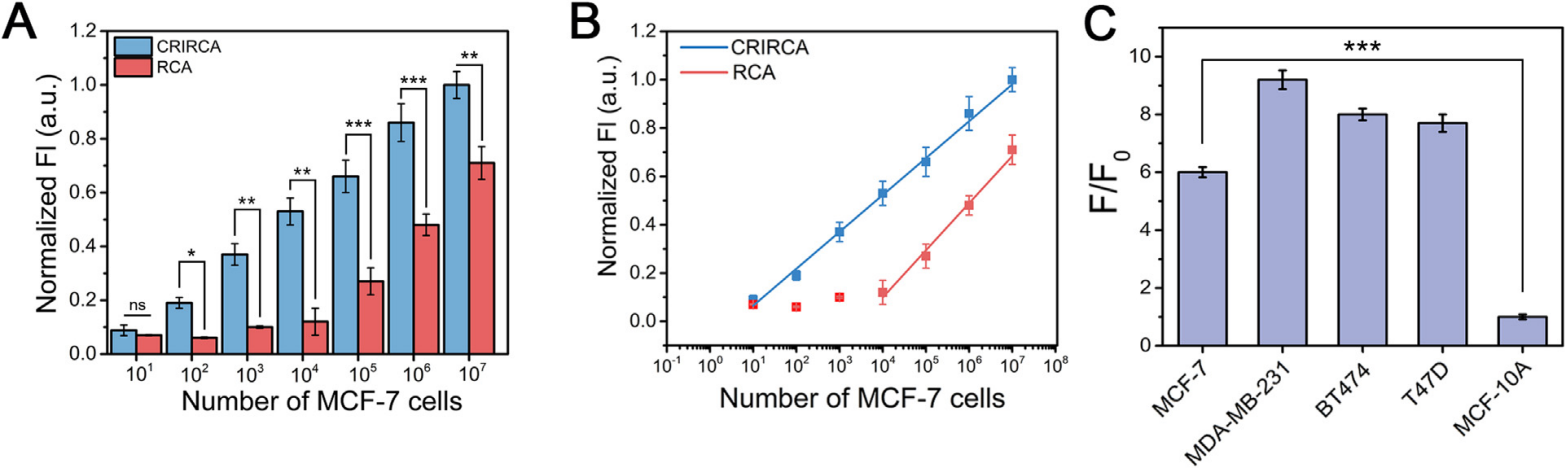
Quantitative investigation of CRIRCA. (A) Fluorescence intensity of amplified products of CRIRCA and RCA from different number of cells. (B) Linear relationship between the fluorescence and the logarithm of cell amount. (C) Fluorescence signal-to-noise ratio of different cells (4 × 10^7^) using CRIRCA. The error bars in the data indicate the standard deviations calculated from three replicate experiments (n = 3, ***p < 0.001, **p < 0.01, *p < 0.05, n.s. means statistically nonsignificant).

Due to the varying sizes and molecular weights of ecDNA obtained by extraction, we investigated the sensitivity of CRIRCA through analyzing ecDNA from different cell numbers. Fluorescence signals of the amplification products of CRIRCA and RCA were measured by preparing a series of 10-fold dilutions from 10^7^ to 10 MCF-7 cells. As shown in Fig. 4A, it was clear that the fluorescence signals increased with the gradual increase of cell numbers. Of note, the fluorescence signal of RCA significantly increased when the cell number was more than 10^3^ and the linear equation is Y = 0.19493X-0.68052 (R^2^ = 0.991), with a detection limit of 1152 cells. Compared with RCA, CRIRCA showed a certain downward trend in fluorescence signals below 100 cells, with a linear equation of Y = 0.15295X-0.0874 (R^2^ = 0.992) and a detection limit of about 1 cell (Fig. 4B). The results indicated that CRIRCA exhibited good sensitivity compared with RCA, which is expected to achieve the detection limit at the single-cell level. Meanwhile, the electrophoresis results also showed that the amplified product bands of CRIRCA were brighter than those of RCA, which suggested that CRIRCA has higher amplification efficiency than RCA (Fig. S7). The notably low LOD in the CRIRCA method attributes to the effective exponential amplification, which is a process driven by the combined actions of Cas9n and DNA polymerase that facilitate repeated cycles of priming, extension, nicking, and strand displacement. Next, we evaluated the feasibility of CRIRCA for ecDNA detection from four types of breast cancer cells (BT474, MDA-MB-231, MCF-7, T47D) and normal breast cells (MCF-10A). As shown in Fig. 4C, the fluorescence signal-to-noise ratio of breast cancer cells were significantly higher than MCF-10A, indicating that the content of ecDNA in cancer cells is much higher than that in normal cells. In addition, the fluorescence signal-to-noise ratio of MCF-7, MDA-MB-231, BT474, and T47D cells varies among tumor cells, indicating that the content of ecDNA varies among different tumor cell lines. This difference may provide a theoretical basis for the clinical diagnosis and identification of cancer with ecDNA.

### Gene identification of ecDNA

**Fig. 5:**
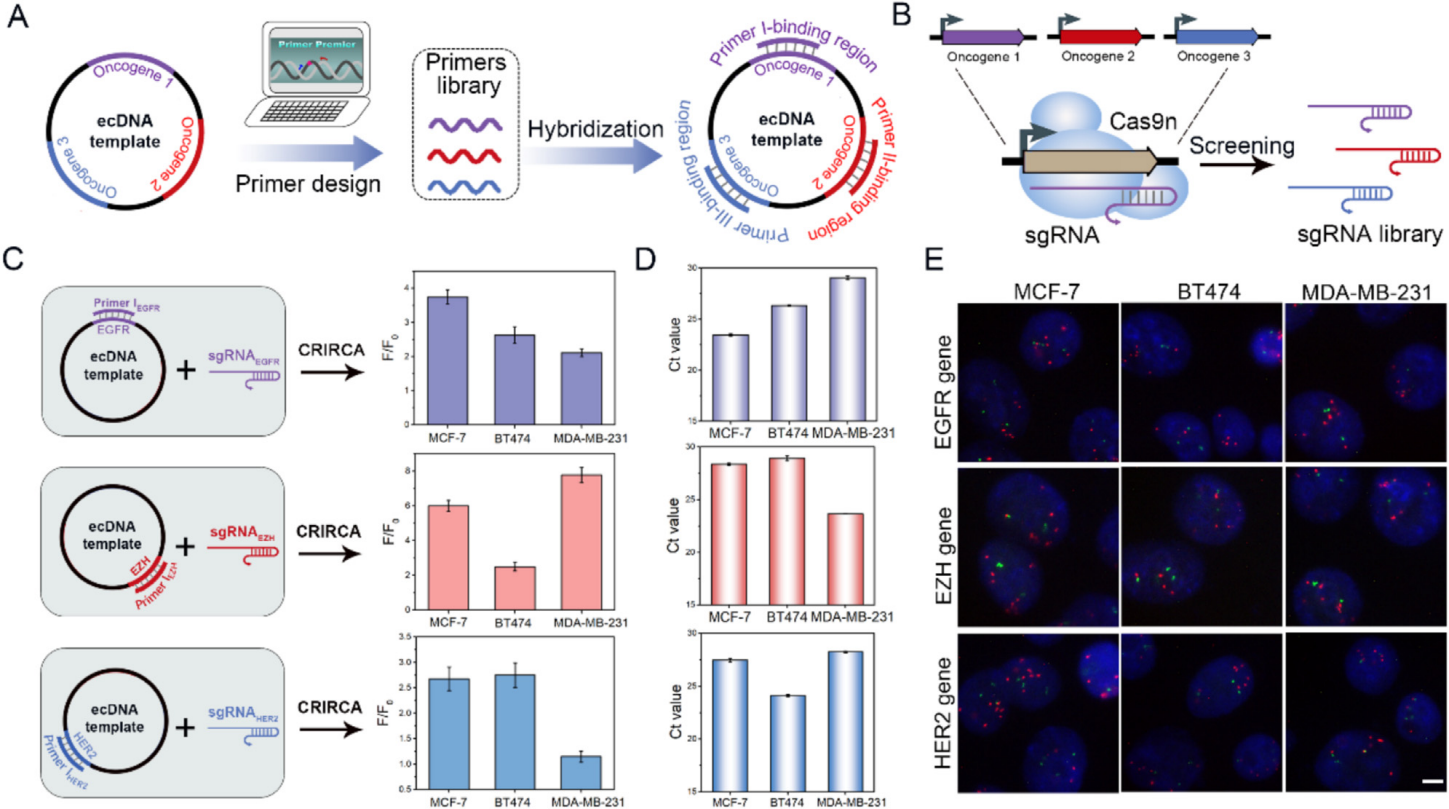
Gene identification of ecDNA. (A-B) Construction of ecDNA gene primer library and sgRNA library. (C) Gene identification was performed on ecDNA of different tumor cell lines by CRIRCA. (D) qPCR identification of three oncogenes on ecDNA of different tumor cell lines. The error bars in the data indicate the standard deviations calculated from three replicate experiments. (E) FISH characterization of oncogenes in breast cancer cell genomes. Red signal indicates the location of oncogenes, green signal represents the position of the centromere. Scale bars, 5 μm. (For interpretation of the references to colour in this figure legend, the reader is referred to the web version of this article.)

ecDNA contains complete oncogenes and drives oncogene expression. Therefore, it is necessary to identify the oncogenes on ecDNA for the clinical diagnosis of ecDNA. In order to evaluate specific oncogenes of ecDNA, we constructed a gene primer library of breast cancer cells (MCF 7, MDA-MB-231 and BT474). As shown in Fig. 5A, using computer-aided design, we employed the Primer Premier 6.0 software to design the upstream and downstream primers of CRIRCA. These primers, which bind to ecDNAs, were designed according to the sequence of different oncogenes to form the gene primer library. Moreover, corresponding sgRNA sequences were designed in parallel with the primer library, utilizing the same computational approach to ensure consistency in targeting the functional regions of oncogenes. These sgRNAs were designed to align with the upstream and downstream primers of CRIRCA and transcribed *in vitro* to construct the sgRNA library (Fig. 5B).

Then CRIRCA was used to identify the genes on ecDNAs in breast cancer cell lines (MCF-7, BT474 and MDA-MB-231) based on designed gene library primer. As shown in Fig. 5C, the fluorescence signal-to-noise ratio of EGFR, EZH and HER2 genes varied in three cancer cells, indicating the three oncogenes had differential expression on ecDNAs in MCF-7, BT474 and MDA-MB-231 cells. In addition, to ensure that the occurrence of CRIRCA is induced by gene primers, non-targeting ecDNA gene primers were adopted to evaluate the specificity of CRIRCA. We have designed primers based on Bacillus thuringiensis (Bt) gene, which theoretically does not exist in human cancer cells. The results showed that the fluorescence signal to noise was close to 1, indicating that Bt gene was absent on ecDNA in the three breast cancer cells (Fig. S8). At the same time, we used qPCR to verify the above CRIRCA results, and the results of qPCR are almost consistent with those of CRIRCA (Fig. 5D). Finally, FISH probes that can label double stranded DNA to label the corresponding oncogene sequence in the nucleus. According to the number and location of red signals (green signals represent centromeres), the expression of each oncogene in each cell indicated that the oncogene expression of EGFR, EZH and HER2 on chromosome was consistent with that on ecDNA in three cancer cells (Fig. 5E). Collectively, it should be noted that genes present on the ecDNA can amplify corresponding signals according to their content, demonstrating that CRIRCA has high accuracy and specificity in gene identification.

## Conclusions

In summary, we have developed a novel nucleic acid amplification technique, called CRIRCA, which utilizes the advantages of high specificity of CRISPR technology and high sensitivity of RCA technology. On the basis of extracting ecDNA, this work successfully characterized the circular structure of ecDNA by AFM. Using ecDNA directly as a circular template, we can employ isothermal amplification to distinguish ecDNA from linear chromosomal DNA based on the presence of disperse electrophoretic bands, which indirectly provides valuable information about the circular structure of the ecDNA. Compared to traditional RCA methods, CRIRCA has achieved significantly enhanced signal amplification, which enables sensitive analysis of even small quantities of ecDNA from tumor cells. The quantitative analysis results revealed that cancer cells contain significantly higher levels of ecDNA compared to normal cells. Additionally, there are discernible variations in ecDNA content between different cancer cell types, which underscore the potential of CRIRCA in providing valuable insights into the role of ecDNA in cancer biology. By employing computer-aided design, we have successfully constructed the primer library and sgRNA library targeting oncogene in ecDNA, and adopted CRIRCA technology to identify the oncogenes of ecDNA in breast cancer cells. This approach is expected to simultaneously acquire structural, sequence, and quantitative information on ecDNA, which will fill the gap in early cancer monitoring research targeting ecDNA and provide support for precise diagnosis and treatment of cancer.

However, challenges remain in evaluating the application value of CRIRCA technology to analyze ecDNA in cancer clinical diagnosis. First, CRIRCA does not offer full-length sequencing of ecDNA, especially for larger species. Therefore, integrating long-read sequencing technologies with optical mapping would be extremely beneficial [40]. Moreover, since CRIRCA measures ecDNA abundance rather than direct transcriptional outputs, integrating complementary approaches such as RNA-seq or ATAC-seq could provide a more comprehensive view of ecDNA-driven oncogene expression. Finally, we have mainly used established breast cancer cell lines to verify the feasibility of the CRIRCA method at present. In the future, patient-derived ecDNA will be applied to evaluate the clinical applicability of CRIRCA comprehensively.

## Compliance with Ethics Requirements

*This article does not contain any studies with human or animal subjects*.

## CRediT authorship contribution statement

**Yuchen Song:** Conceptualization, Methodology, Writing – original draft. **Chaoyang Guan:** Investigation, Data curation. **Yue Zhang:** Investigation, Data curation. **Yiming Xu:** Investigation, Data curation. **Pengfei Li:** Formal analysis, Visualization. **Liqiang Luo:** Resources. **Chang Feng:** Writing – review & editing. **Guifang Chen:** Supervision, Funding acquisition.

## Declaration of competing interest

*The authors declare that they have no known competing financial interests or personal relationships that could have appeared to influence the work reported in this paper*.

## Data Availability

Data will be made available on request.
