# Supplementary material for: A novel CRISPR-Cas9 nickase-mediated rolling circle amplification (CRIRCA) technique for gene identification and quantitative analysis of extrachromosomal DNA
Source: J Adv Res. 2025 Apr 22;80:239–48. doi: 10.1016/j.jare.2025.04.031 (PMC12869246; doi:10.1016/j.jare.2025.04.031)
Supplement: Supplementary Data 1 [file mmc1.docx]

**SUPPLEMENTARY INFORMATION**

**A novel CRISPR-Cas9 nickase-mediated rolling circle amplification (CRIRCA) technique for gene identification and quantitative analysis of extrachromosomal DNA**

Yuchen Song^a,b^, Chaoyang Guan^a,d^, Yue Zhang^c^, Yiming Xu^c^, Pengfei Li^c^, Liqiang Luo^b,^*, Chang Feng^a,d,^*, Guifang Chen^a,d,^*

^a^ Center for Molecular Recognition and Biosensing, Shanghai Engineering Research Center of Organ Repair, Joint International Research Laboratory of Biomaterials and Biotechnology in Organ Repair (Ministry of Education), School of Life Sciences, Shanghai University, Shanghai 200444, P. R. China

^b^ Department of Chemistry, College of Sciences, Shanghai University, Shanghai 200444, P. R. China.

^c^ Department of Applied Biology, East China University of Science and Technology, Shanghai 200237, P. R. China.

^d^ Shanghai Key Laboratory of Bio-Energy Crops, School of Life Sciences, Shanghai University, Shanghai 200444, P. R. China

*Corresponding authors: luck@shu.edu.cn; [cfeng@shu.edu.cn](mailto:cfeng@shu.edu.cn); gfchen@shu.edu.cn

**Table S1.** Oligonucleotides used in this study.

| Oligonucleotide |  | Sequence (5’→3’) |
| --- | --- | --- |
| Epidermal growth factor receptor (EGFR) | Primer 1 | GGCACTTTTGAAGATCATTTTCTC |
|  | Primer 2 | CTGTGTTGAGGGCAATGAG |
|  | qPCR Primer 1 | GGCACTTTTGAAGATCATTTTCTC |
|  | qPCR Primer 2 | ACTTCAAGTGGAATTCTGCC |
| Human epidermal growth factor receptor 2 (HER-2) | Primer 1 | GTGTGGACCTGGATGACAAGGG |
|  | Primer 2 | GCTCCACCAGCTCCGTTTCCTG |
|  | qPCR Primer 1 | GTGTGGACCTGGATGACAAGGG |
|  | qPCR Primer 2 | GAGCAGGTCCTCTGTCACTTGG |
| Enhancer of zeste homolog (EZH) | Primer 1 | AATCAGAGTACATGCGACTGAGA |
|  | Primer 2 | GCTGTATCCTTCGCTGTTTCC |
|  | qPCR Primer 1 | AATCAGAGTACATGCGACTGAGA |
|  | qPCR Primer 2 | CCTAAGCTTCCAAGTATTCACTC |
| Bacillus thuringiensis (Bt) | Primer 1 | GAAGGTTTGAGCAATCTCTAC |
|  | Primer 2 | CGATCAGCCTAGTAAGGTCGT |
| hGluc | Primer 1 | TAATACGACTCACTATAGGGA |
|  | Primer 2 | ATGTATCCGCTCATGAGACAATAACCCTGA |
| EGFR sgRNA template | GTGGAGCCTCTTACACCCAGGTTTTAGAGCTAGAAATAGCAAGTTAAAATAAGGCTAGTCCGTTATCAACTTGAAAAAGTGGCACCGAGTCGGTGC | |
| HER2 sgRNA template | TCATCGCTCACAACCAAGTGGTTTTAGAGCTAGAAATAGCAAGTTAAAATAAGGCTAGTCCGTTATCAACTTGAAAAAGTGGCACCGAGTCGGTGC | |
| EZH sgRNA template | AGAAGGGACCAGTTTGTTGGGTTTTAGAGCTAGAAATAGCAAGTTAAAATAAGGCTAGTCCGTTATCAACTTGAAAAAGTGGCACCGAGTCGGTGC | |
| hGluc sgRNA | GCCGAGGCCAAGCCCACCGAGTTTTAGAGCTAGAAATAGCAAGTTAAAATAAGGCTAGTCCGTTATCAACTTGAAAAAGTGGCACCGAGTCGGTGC | |
| Bt sgRNA template | TGAGTATAGTTGGAGAGACTGTTTTAGAGCTAGAAATAGCAAGTTAAAATAAGGCTAGTCCGTTATCAACTTGAAAAAGTGGCACCGAGTCGGTGC | |

**Table S2.** Construction PCR system designed in this work.

| Component | Amount (μL) |
| --- | --- |
| ddH_2_O | 32.5 |
| 5×PS buffer | 10 |
| PrimeSTAR HS DNA polymerase (Takara) | 0.5 |
| dNTPs (25 mM) | 4 |
| pET28a(+)-Cas9 | 1 |
| Cas9-D10A-F (5 μM) | 1 |
| Cas9-D10A-R (5 μM) | 1 |
| Total | 50 |

Procedure

| Temperature | Time |
| --- | --- |
| 95 ℃ | 5 min |
| 95 ℃ | 30 s |
| 64 ℃ | 30 s 30 cycles |
| 72 ℃ | 90 s |
| 72 ℃ | 10 min |
| 25 ℃ | hold |

**Table S3.** Ligation system designed in this work.

| Component | Amount (μL) |
| --- | --- |
| ddH_2_O | 7 |
| 10×T4 ligation buffer | 1.5 |
| T4 DNA ligase (Takara) | 0.5 |
| pET28a(+)-Cas9 digestion products | 3 |
| Cas9 fragment digestion products | 3 |
| Total | 15 |

*Replace T4 DNA ligase with ddH_2_O as ligation control.

Procedure

| Temperature | Time |
| --- | --- |
| 4 ℃ | overnight |

**Table S4.** Verification PCR system designed in this work.

| Component | Amount (μL) |
| --- | --- |
| ddH_2_O | 7 |
| Premix Taq*^™^* (TaKaRa Taq*^™^* Version 2.0) | 10 |
| Template | 1 |
| T7-pro (5 μM) | 1 |
| Cas-D10A-R (5 μM) | 1 |
| Total | 20 |

*Pick bacteria clones from LB agar medium with pipette tips and immerse tips in 50 μL sterile ddH_2_O to make bacterial suspensions, which are used as template.

Procedure

| Temperature | Time |
| --- | --- |
| 95 ℃ | 5 min |
| 95 ℃ | 30 s |
| 61 ℃ | 30 s 30 cycles |
| 72 ℃ | 90 s |
| 72 ℃ | 10 min |
| 25 ℃ | hold |

**Table S5.** List of primer sequences used for pET28a(+)-nCas9(D10A) construction.

| Primer Name | Primer sequence (5’-3’) |
| --- | --- |
| Cas9-D10A-F | AGCCGGAATTCATGGACAAGAAGTACAGCATCGGC  CTGGCTATCGGCACCAACAGCGTG |
| Cas9-D10A-R | AGTACGGGATCCGGAAGGTCAGGATCTTCT |
| T7-pro | TAATACGACTCACTATAGGG |


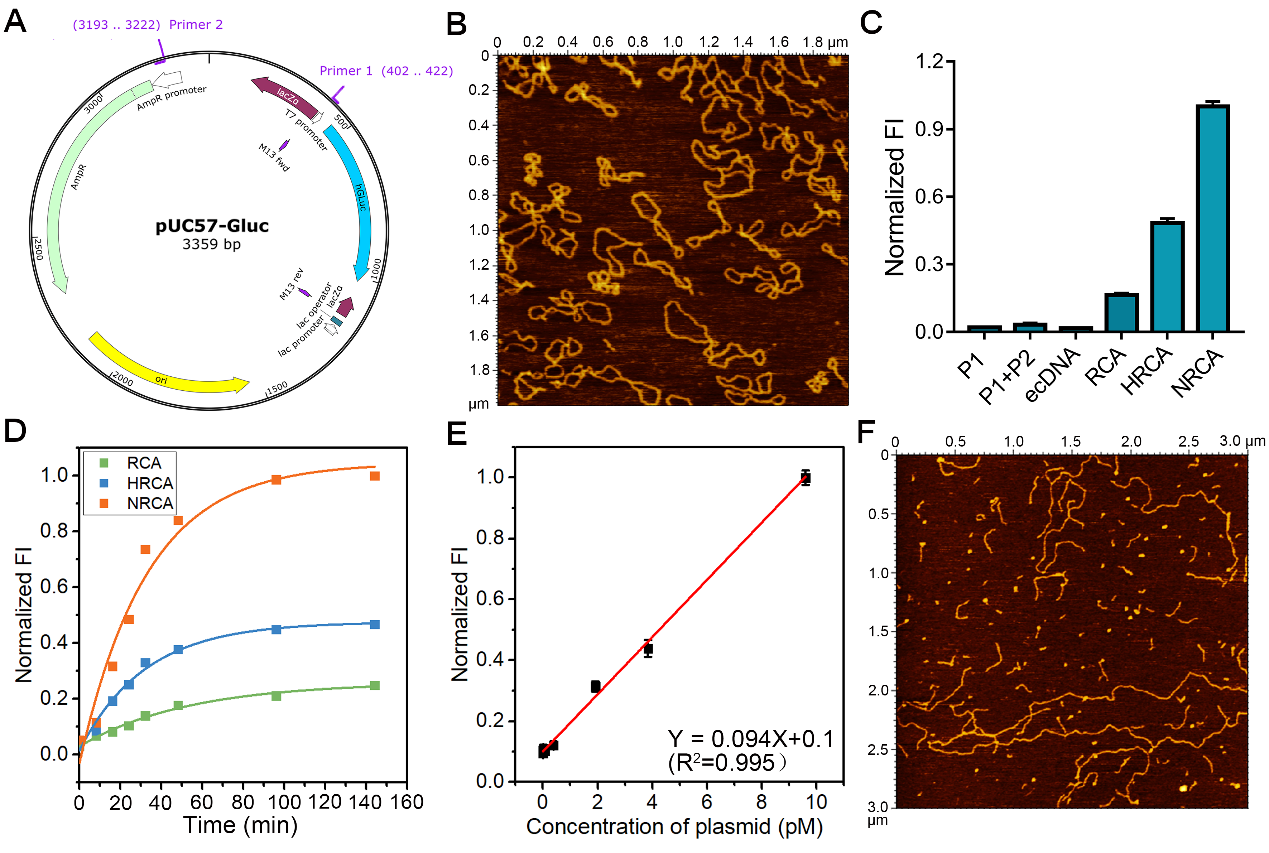


**Fig. S1.** Feasibility analysis of plasmid DNA using NRCA. (A) Schematic diagram of plasmid DNA construction and primer design. (B) AFM characterization of plasmid DNA. (C) The fluorescence signal of RCA, HRCA and NRCA for plasmid DNA detection. (D) The kinetics of RCA, HRCA and NRCA reaction. (E) The linear relationship between concentration of plasmid DNA and fluorescence signal intensity. (F) AFM characterization of plasmid DNA amplified by HRCA.


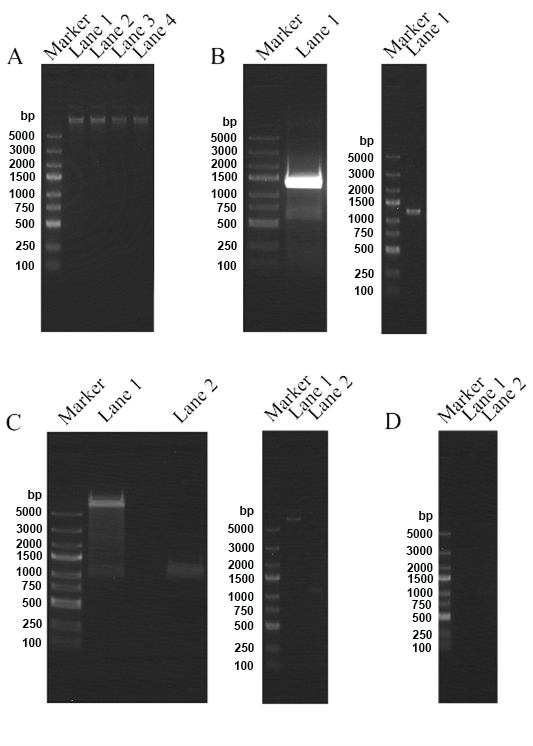


**Fig. S2.** Agarose gel eletrophoresis for pET28a(+)-nCas9(D10A) construction. (A) Vector for construction. Lane 1, Lane 2, Lane 3 and Lane 4 are both pET28a(+)-Cas9. (B) A fragment of Cas9 (1359 bp) amplification, a point mutation is introduced by primer Cas9-D10A-F and Cas9-D10A-R, to change the tenth aspartic acid to alanine (left); gel extraction for amplification products (right). (C) Restriction enzyme digestion for vector and PCR products, Lane1 is pET28a(+)-Cas9 digested by *Eco*R Ⅰ and *Bam*H Ⅰ, and Lane 2 is PCR products digested by *Eco*R Ⅰ and *Bam*H Ⅰ (left); gel extraction for restriction enzyme digestion products (right). (D) Ligation for vector and PCR products, Lane 1 is ligation products, Lane 2 is ligation control. The markers are 250 bp-Ⅱ DNA ladder.


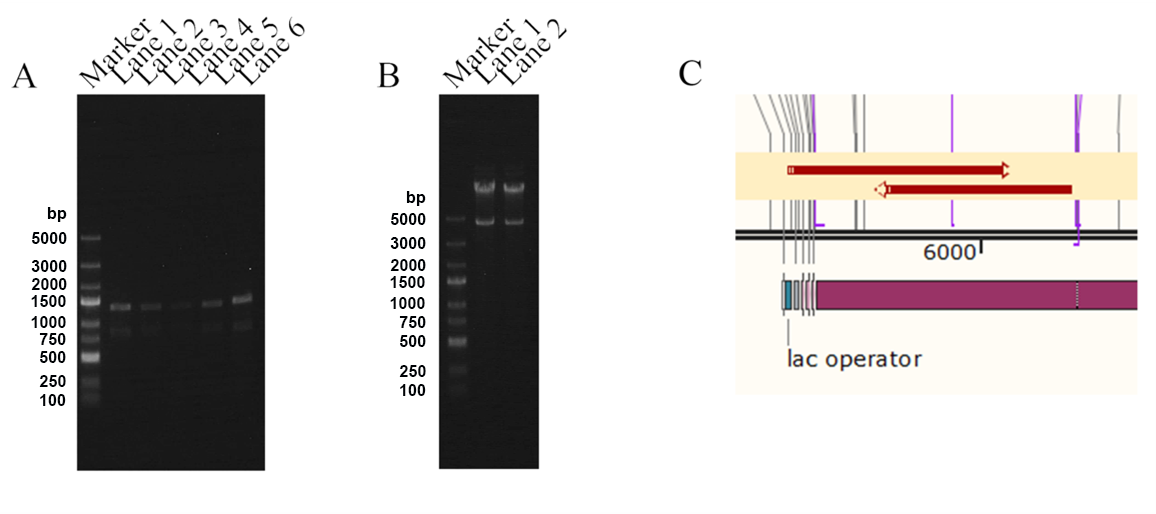


**Fig. S3.** Agarose gel eletrophoresis for pET28a(+)-Cas9n(D10A) verification. (A) Lane 1~6 are transformant identification PCR with primer Cas9-D10A-R and T7-pro primer. (B) Extraction of positive transformant plasmids, Lane 1 and Lane 2 are both recombinant plasmids. (C) Recombinant plasmid sequencing. The markers are 250 bp-Ⅱ DNA ladder.


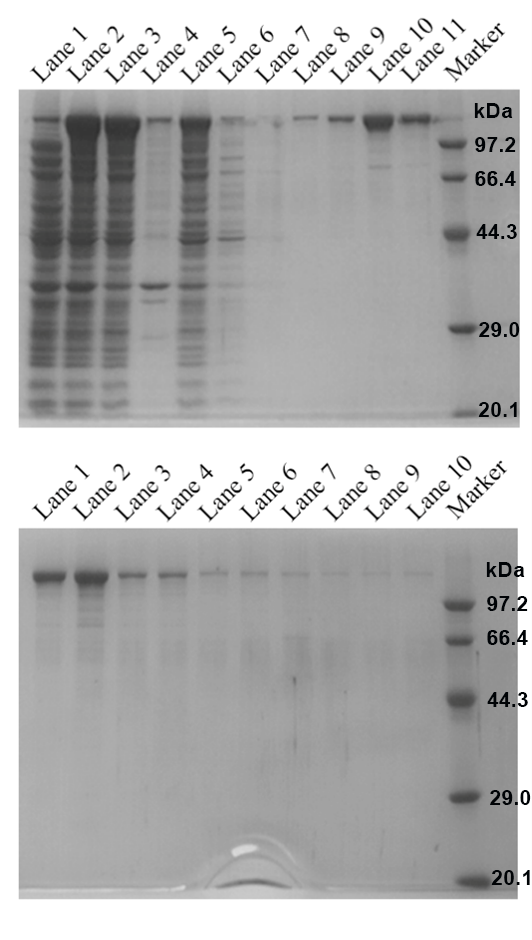


**Fig. S4.** SDS-PAGE for Cas9n(D10A) expression and purification. Lane 1: whole proteins before IPTG induction, Lane 2: whole proteins after IPTG induction, Lane 3: supernatant of ultrasonic disruption, Lane 4: precipitation of ultrasonic disruption, Lane 5: affinity chromatography flow-through products, Lane 6: affinity chromatography wash products, Lane 7: 20 mM imidazole elution products, Lane 8: 50 mM imidazole elution products, Lane 9~11: 100 mM imidazole elution products (up); Lane 1~3: 200 mM imidazole elution products, Lane 4~6: 300 mM imidazole elution products, Lane 7~9: 400 mM imidazole elution products, Lane 10: 500 mM imidazole elution products (down). The markers are Premixed Protein Marker (Low) (Takara).


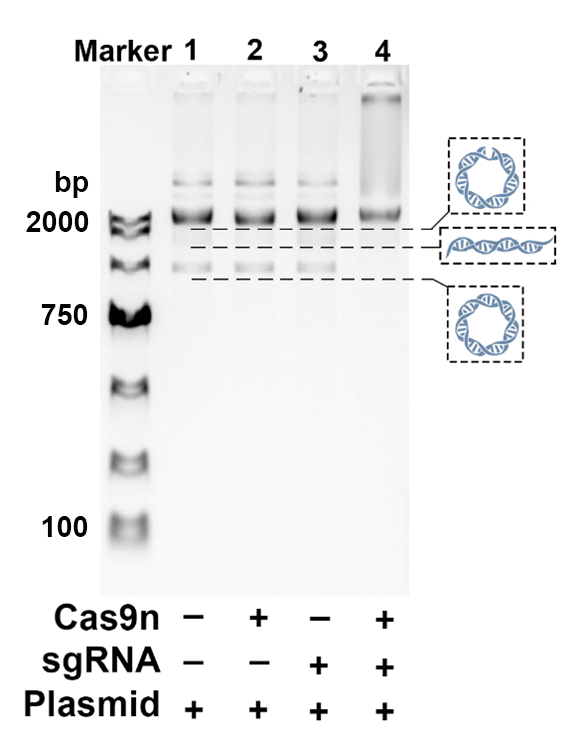


**Fig. S5.** Electrophoresis characterization of pUC57 plasmid cleavage by sgRNA:Cas9n complexes.


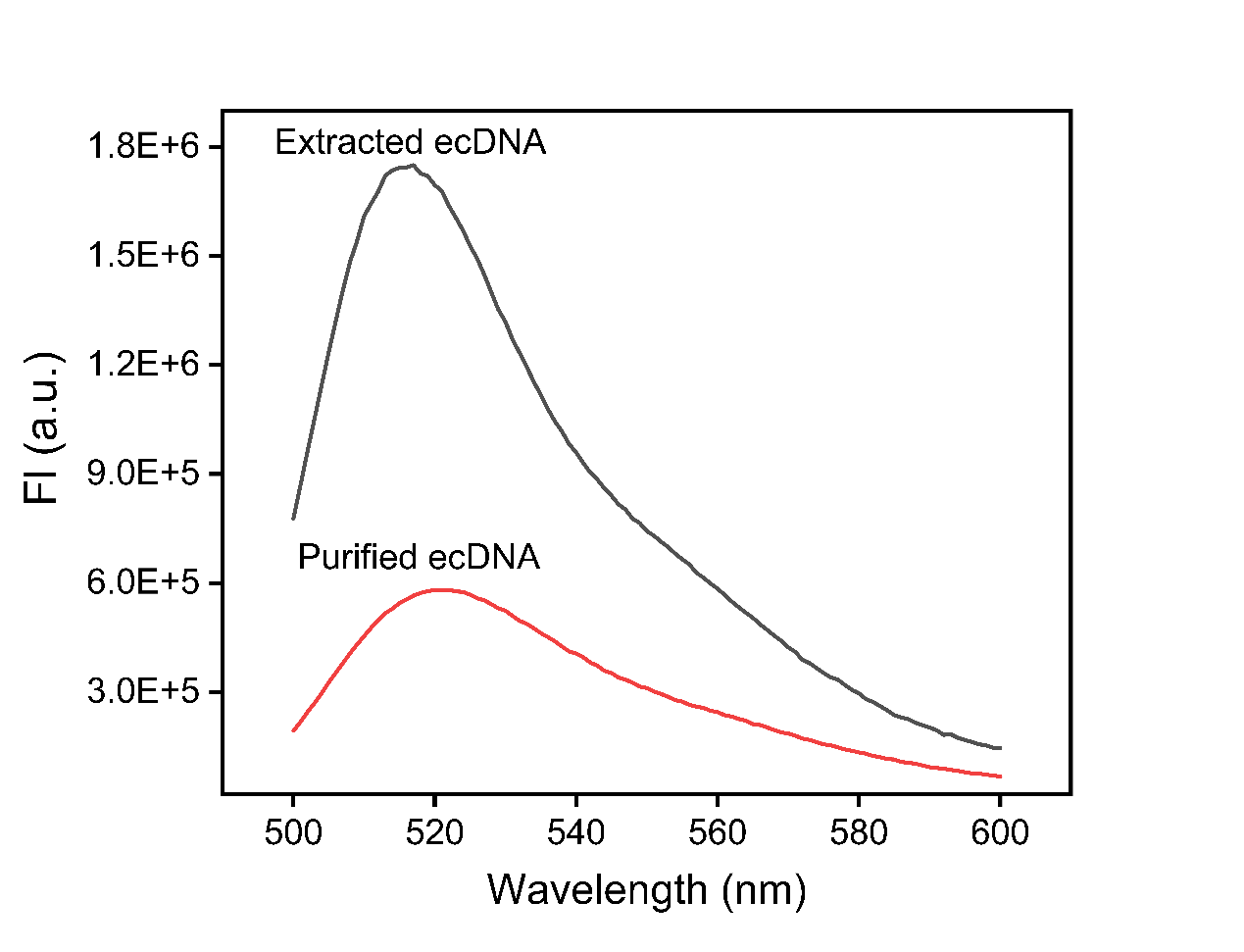
**Fig. S6.** Fluorescence characterization of extracted ecDNA and purified ecDNA by CRIRCA.


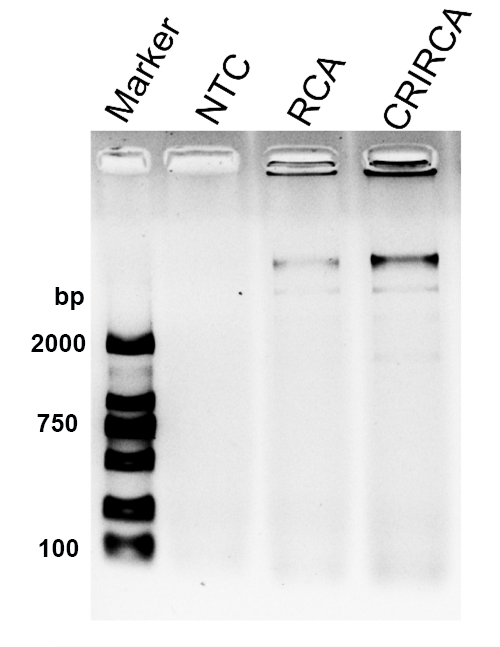


**Fig. S7.** Electrophoresis characterization of RCA and CRIRCA products.


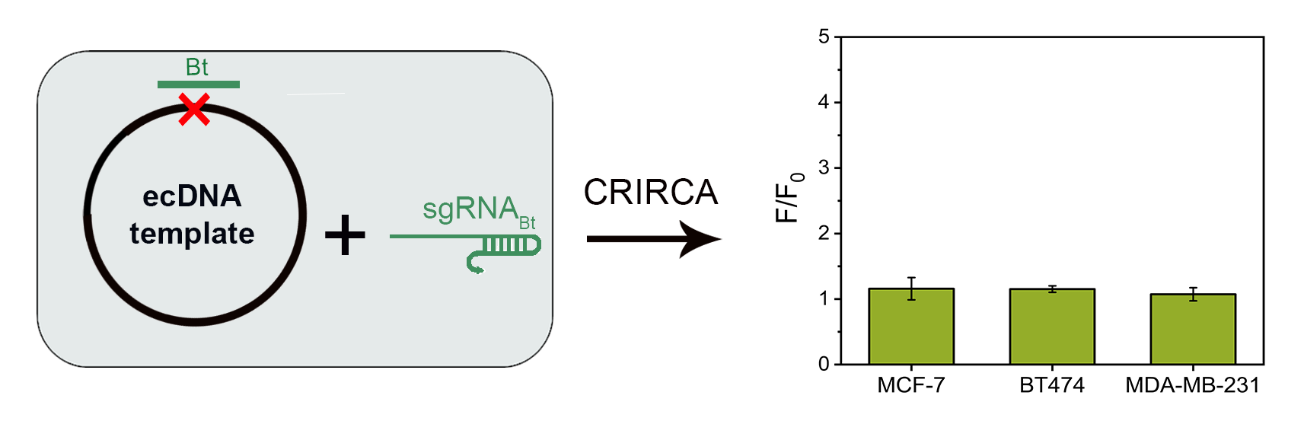


**Fig. S8.** Negative gene identification was performed on ecDNA of different tumor cell lines by CRIRCA. Error bars represent standard deviations from three replicates.
